# The evolution of NLRC3 subfamily genes in Sebastidae teleost fishes

**DOI:** 10.1186/s12864-023-09785-5

**Published:** 2023-11-14

**Authors:** Chengbin Gao, Xin Cai, Alan J. Lymbery, Le Ma, Chao Li

**Affiliations:** 1 School of Marine Science and Engineering, Qingdao Agricultural University, 266109 Qingdao, China; 2 Centre for Sustainable Aquatic Ecosystems, Harry Butler Institute, Murdoch University, 6150 Murdoch, WA Australia

**Keywords:** Immune system, Whole-genome duplication, Gene expansion and contraction, Phylogenetics, Bacterial Infection

## Abstract

**Background:**

With more than 36,000 valid fish species, teleost fishes constitute the most species-rich vertebrate clade and exhibit extensive genetic and phenotypic variation, including diverse immune defense strategies. NLRC3 subfamily genes, which are specific to fishes, play vital roles in the immune system of teleosts. The evolution of teleosts has been impacted by several whole-genome duplication (WGD) events, which might be a key reason for the expansions of the NLRC3 subfamily, but detailed knowledge of NLRC3 subfamily evolution in the family Sebastidae is still limited.

**Results:**

Phylogenetic inference of NLRC3 subfamily protein sequences were conducted to evaluate the orthology of NLRC3 subfamily genes in black rockfish (*Sebastes schlegilii*), 13 other fish species from the families Sebastidae, Serranidae, Gasterosteidae and Cyclopteridae, and three species of high vertebrates (bird, reptile and amphibian). WGD analyses were used to estimate expansions and contractions of the NLRC3 subfamily, and patterns of expression of NLRC3 subfamily genes in black rockfish following bacterial infections were used to investigate the functional roles of these genes in the traditional and mucosal immune system of the Sebastidae. Different patterns of gene expansions and contractions were observed in 17 fish and other species examined, and one and two whole-genome duplication events were observed in two members of family Sebastidae (black rockfish and honeycomb rockfish, *Sebastes umbrosus*), respectively. Subsequently, 179 copy numbers of NLRC3 genes were found in black rockfish and 166 in honeycomb rockfish. Phylogenetic analyses corroborated the conservation and evolution of NLRC3 orthologues between Sebastidae and other fish species. Finally, differential expression analyses provided evidence of the immune roles of NLRC3 genes in black rockfish during bacterial infections and gene ontology analysis also indicated other functional roles.

**Conclusions:**

We hypothesize that NLRC3 genes have evolved a variety of different functions, in addition to their role in the immune response, as a result of whole genome duplication events during teleost diversification. Importantly, this study had underscored the importance of sampling across taxonomic groups, to better understand the evolutionary patterns of the innate immunity system on which complex immunological novelties arose. Moreover, the results in this study could extend current knowledge of the plasticity of the immune system.

**Supplementary Information:**

The online version contains supplementary material available at 10.1186/s12864-023-09785-5.

## Background

Ray-finned teleost fishes occupy the vast majority of the more than 36,000 currently described fish species [1]. Over their 400 million years of evolutionary history [2, 3], teleosts have experienced three rounds of whole-genome duplications (WGD) [4, 5], one more than other vertebrates [6]. Many duplicated genes may lose one of the duplicates via pseudogenization by the accumulation of deleterious mutations during or after duplication events [7–9]. Populations of a species that lose different copies of a duplicated gene may become genetically isolated, according to the divergent resolution model of speciation [10, 11]. Duplicated genes might also be retained, rather than lost, and acquire novel functions [12]: this has been suggested as a driving force for major evolutionary transitions, although the evidence for this is mixed [11, 13].

The adaptive immune system is found in all jawed vertebrates [14, 15], but has undergone fundamental modifications of the immune gene repertoire [16–18]. Through the loss or doubling of key immune genes during or after WGD events, the immune response system of vertebrate species may have evolved compensatory mechanisms in both adaptive and innate immunity, especially for teleost fishes that contain a number of specific genes because of their unique living environments.

The functional roles of NOD-like receptors (NLRs), key components of the innate and adaptive immune system in invertebrate and vertebrate species, have attracted much research attention. In mammals, there are 20–30 NLR family members [19, 20], while larger numbers of NLR member repertoires have been identified in fish species and other early-diverging metazoans [21, 22]. Based on different types of structural domains, NLRs are usually divided into four subfamilies; NLRA, NLRB, NLRC and NLRP [23]. NLRA subfamilies have been well characterized in teleost fishes including grass carp (*Ctenopharyngodon idella*) [24], channel catfish (*Ictalurus punctatus*) [25], and miiuy croaker (*Miichthys miiuy*) [26]. NLRB and NLRC subfamilies have been also identified preliminarily in several fish species, such as turbot (*Scophthalmus maximus* L.) [27], miiuy croaker [28] and black rockfish (*S. schlegelii*) [29]. Meanwhile, the important roles of NLRC genes were also mentioned in several fish species. It is well known that CD4^+^ T cells were the key component in the immune system, which played as the center in orchestrating adaptive immune responses against pathogenic infections [30, 31]. In Nile tilapia (*Oreochromis niloticus*), NLRC genes were concentratedly detected in T cells, especially for NLRC3 gene that was mainly observed a high expression level in CD4^+^ T Cell during LPS/LTA stimulation [32], which might suggest the potential functional roles of NLRC3 gene in teleost adaptive immune response.

According to their physiological functions, NLRs can also be classified into three subgroups; inflammasome-forming, reproductive and regulatory NLRs [33]. Several members of the inflammasome-forming NLRs have been well studied and found to cooperate with the maturation of IL-1β and IL-18 to process pyroptosis [34]. Moreover, regulatory NLRs may perform important functions by acting as either positive or negative regulators on several immune signaling pathways, including the NF-kB and mitogen-activated protein kinase (MAPK) signaling pathway, the type I IFN response and the NOD1-RIPK2 antibacterial pathway [33, 35–37]. In fish, regulatory NLRs, including the piscine NLRC3 subfamily proteins, have been also identified and their immune functions investigated [38, 39]. For example, in rainbow trout (*Oncorhynchus mykiss*), high expression level of NLRC3 gene was induced in the immune tissues and gill cells overexpressed proinflammatory cytokines in response to Poly (I:C) stimulation [40]. Similarly, significant up-regulations of NLRC3 alongside with significantly up-regulated inflammatory cytokines were detected in the cells from different immune tissue of Asian seabass (Acipenser baerii) after Poly(I:C) stimulation, which might indicate that NLRC3 genes play a critical intracellular pattern recognition receptor to respond to viral infection in fish species [41]. Meanwhile, more studies were conducted on the antibacterial roles of NLRC3 genes in different teleost species, which explored and identified the significantly differential expressions (mainly in up-regulations) of piscine NLRC3 genes during bacterial infections [40, 42–44]. However, knowledge of the causes of NLR copy number variation and their functions in the immune system, especially for the evolution of the regulatory, fish-specific NLRC3 subfamily proteins, is still limited in teleost fishes.

In this study, the fully assembled genome of 17 species, including chicken, turtle, frog and 14 fish species (including eight members of the order Perciformes), were selected for comparative analyses. First, the presence or absence of key genes in the vertebrate adaptive immune response system were examined in the 14 fish species and three high vertebrates to infer potential compensatory mechanisms of lost or doubled gene repertoires. Second, the copy number of the piscine NLRC3 gene repertoire was calculated and compared among species. Next, phylogenetic inference of NLRC3 subfamily protein sequences was conducted to evaluate the orthology of NLRC3 subfamily genes between black rockfish (*S. schlegilii*) and other species. Finally, gene expressions of piscine NLRC3 gene repertoires were analyzed in different tissues of black rockfish following bacterial infections to infer their key roles in the immune response system of this species. The results of this study highlight the plasticity of the vertebrate innate and adaptive immune system and support one of the main roles of piscine NLRC3 genes, ‘key immune antibacterial genes’, promoting rapid diversification of the immune response system in teleost fishes.

## Results

### Expansions and contractions of gene family

**Fig. 1:**
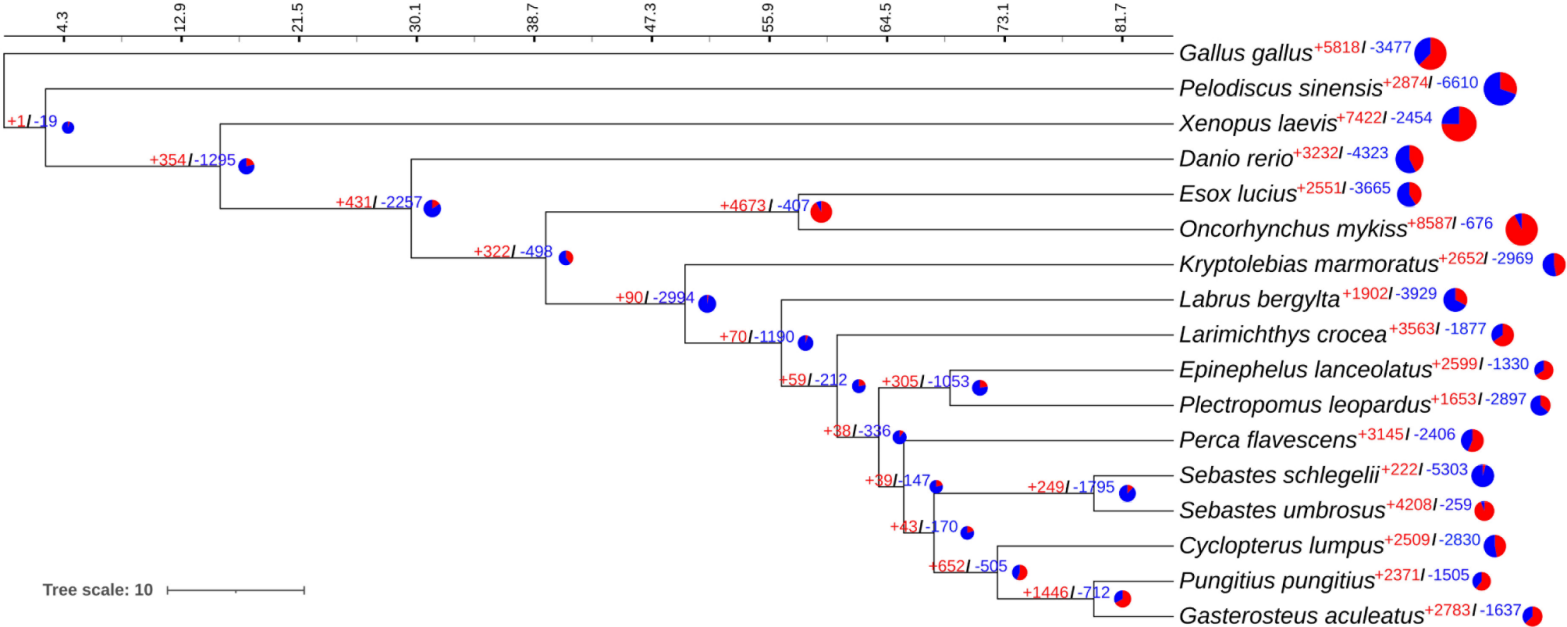
Expansions and contractions of gene families in 17 species including chicken, turtle, frog and 14 teleost species. Gene expansions and contractions assessed by regressing gene counts at internal (ancestral) vs. external (extant) nodes, and the expansions and contractions within each gene family with *P*-value > 0.05 were assessed as statistically significant. Colors in red represent expansions and blue represent contractions

The final maximum likelihood phylogeny of 17 species (including bird, reptile, amphibian and 14 fish species) was constructed, based on the best-fit substitution model, and had a maximum possible lambda value of 0.0116948 (Fig. 1). A total of 13,957 gene families were detected in all genomes, among which 1,609 gene families had significant variance in expansions and contractions. As shown in Fig. 1, in most species, gene family expansions outnumbered contractions. In black rockfish, however, contractions of gene families (5,303) were much greater than expansions (222), while there was a reverse situation (with 4,208 expansions and 259 contractions) in the closely related honeycomb rockfish. Expansions and contractions of the NLRC3 subfamily occurred in almost all teleost species.

**Fig. 2:**
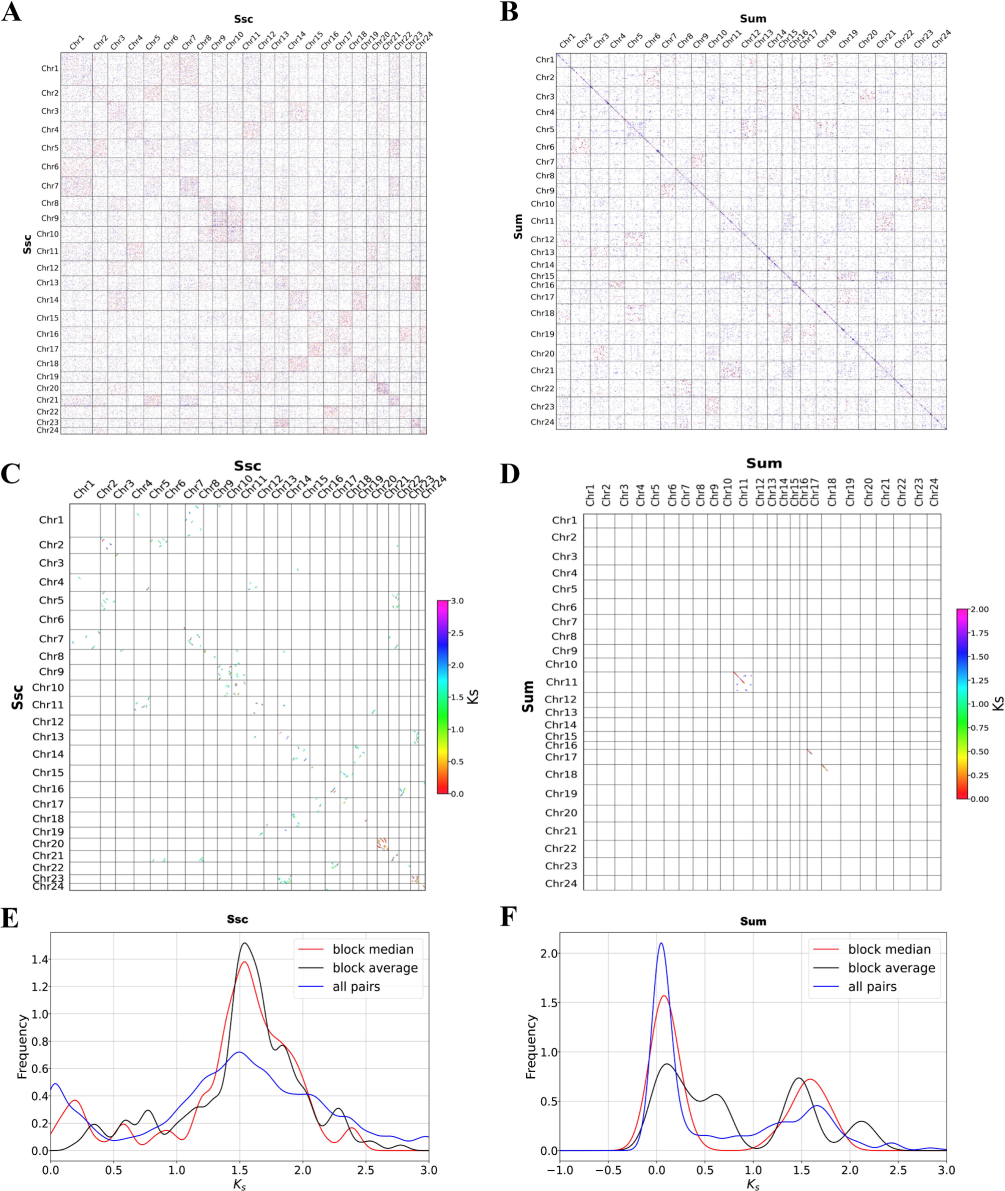
Collinearity analyses of black rockfish and honeycomb rockfish. (**A** and **B**) Dot plots of black rockfish and honeycomb rockfish, respectively. (**C** and **D**) Ks dot plots of black rockfish and honeycomb rockfish, respectively. Dot plots were colored by the value of Ks, with Ks increasing y from red to purple. (**E** and** F**) Frequency map of Ks in black rockfish and honeycomb rockfish, respectively

To further investigate the expansions of the NLRC3 subfamily in Sebastidae, whole genome duplication analyses were conducted for black rockfish and honeycomb rockfish (Fig. 2). First, homologous dot plots indicated that several blue dots were less concentrated than red dots, caused by γ-WGD events (Fig. 2A and B). Second, the Ks dot plots of black rockfish (Fig. 2C) and honeycomb rockfish (Fig. 2D) found Ks values ranging from 0.0 to 3.0 for black rockfish and from 0.0 to 2.0 for honeycomb rockfish. Finally, the Ks peak values were estimated using the Ks values of paralogous genes in black rockfish (Fig, 2E) and honeycomb rockfish (Fig. 2F). For black rockfish, one peak value was found at approximately 1.5, while there were two peak values at about 0.0 and 1.5 for honeycomb rockfish. The results suggested that one WGD event occurred in both black rockfish and honeycomb rockfish, while one more WGD event occurred in honeycomb rockfish. Overall, 68 and 29 NLRC3 genes were observed in the duplication events in honeycomb rockfish and black rockfish, respectively.

### Expansions of NLRC3 genes

**Fig. 3:**
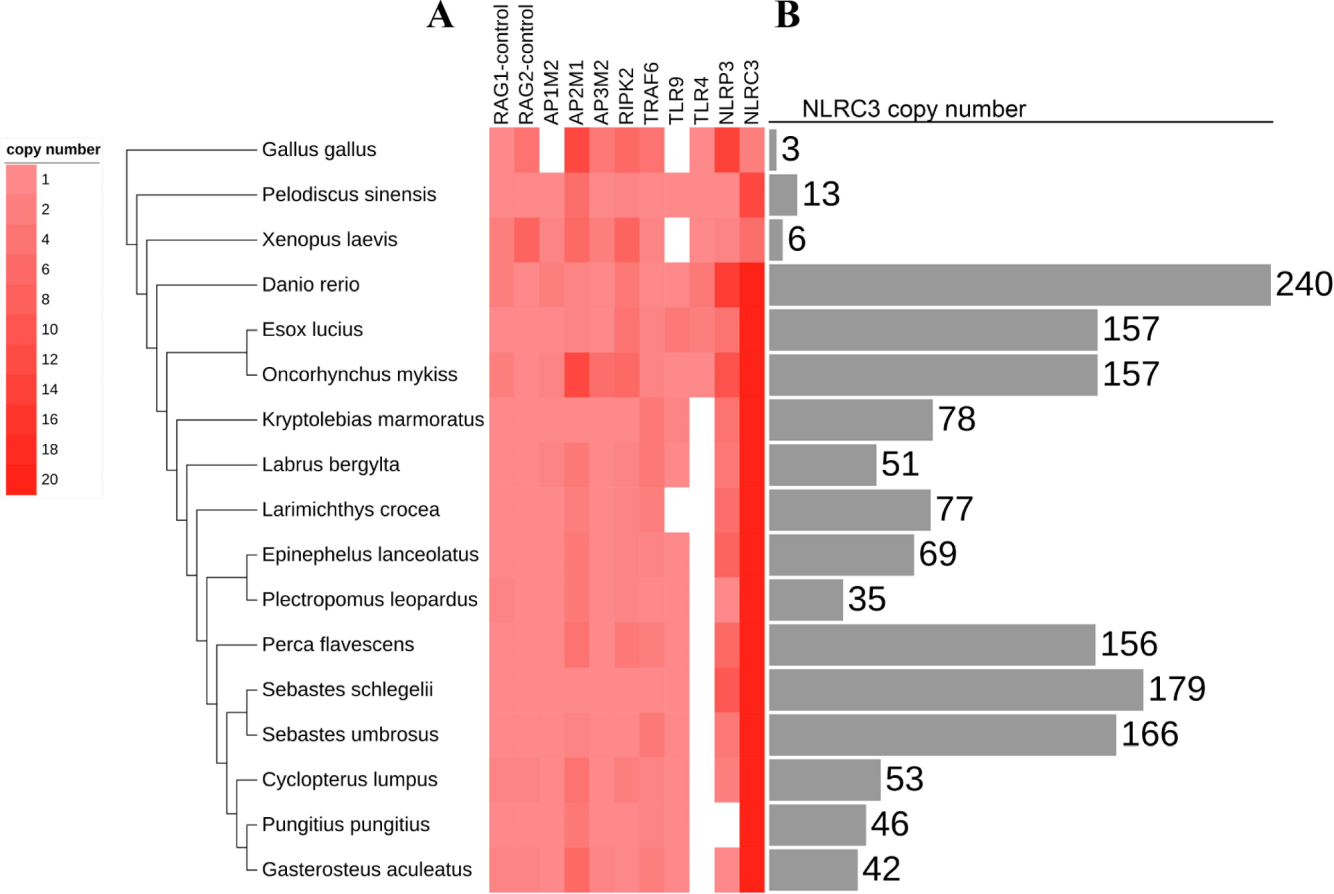
The copy number of immune gene repertoires and estimated NLRC3 genes in the genome of 17 species. **(A)** Presence of key immune genes, and genes interacting with NLRC3 genes. Genes not detected are indicated by white squares. Colors reflect different copy numbers (see key). **(B) **Copy number estimates for NLRC3 genes

The copy numbers of 9 immune gene repertoires and estimated NLRC3 genes (including their alternative splicing sequences), as well as RAG1 and RAG2 genes, in the genome of 17 species are shown in Fig. 3. The highly conserved control genes (RAG1 and RAG2) were identified in all species, while most of the nine selected immune genes were also observed in all species, except for the TLR9 and TLR4 genes (Fig. 3). Moreover, NLRC3 genes were expanded in all teleost species, with extreme expansions were found in zebrafish, followed by members of the Sebastidae family. The copy numbers of NLRC3 genes in black rockfish and honeycomb rockfish were 179 and 166, respectively.

### Phylogenetic analyses of NLRC3 subfamily

**Fig. 4:**
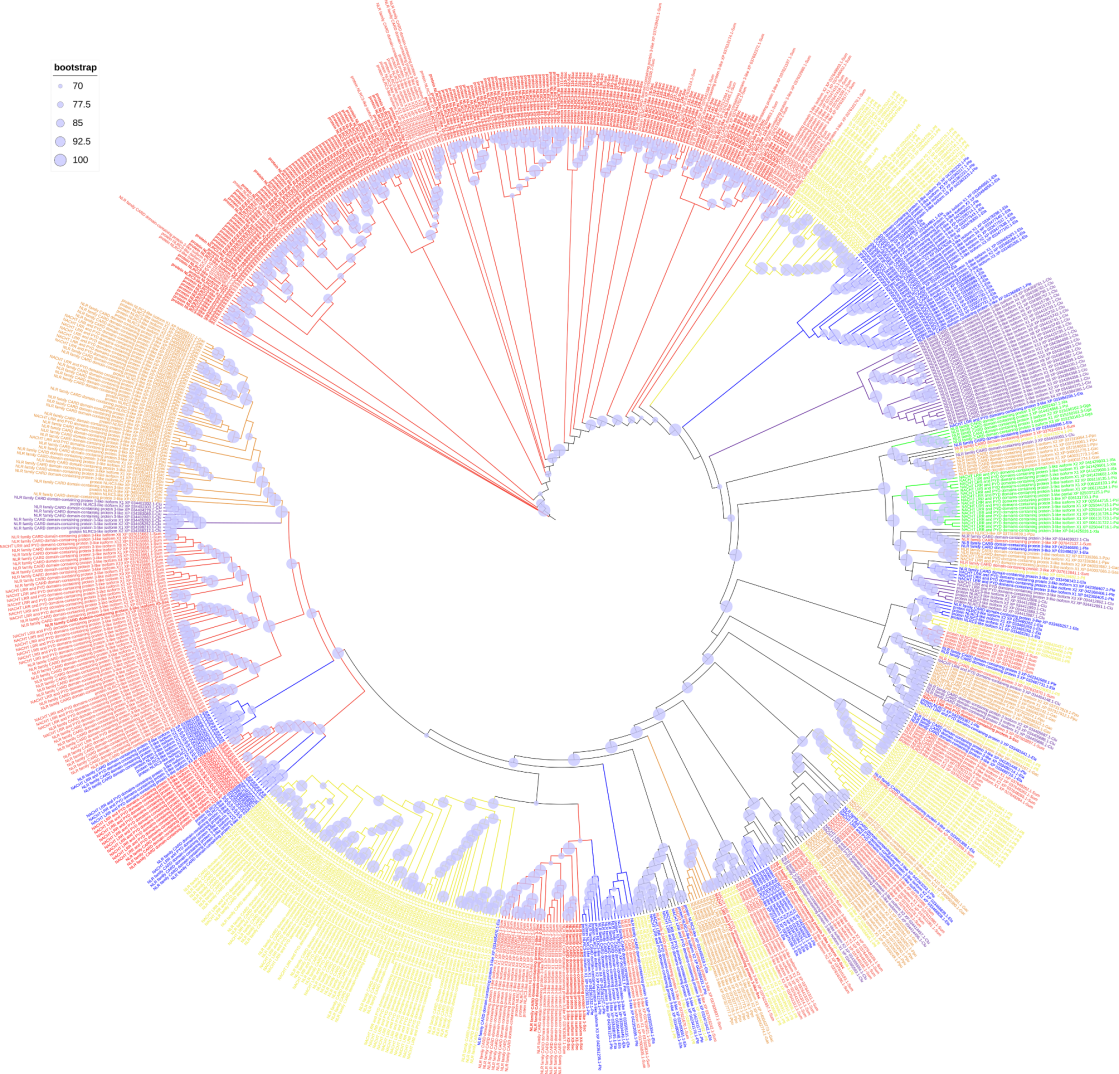
Phylogenetic tree of NLRC3 homologs in Perciformes and high vertebrates. NLRC3 genes of black rockfish were in bold font. Red indicates NLRC3 expansion in the Sebastidae. Yellow, blue, orange and purple indicate NLRC3 expansions of Percidae, Serranidae, Gasterosteidae and Cyclopteridae, respectively, and green for high vertebrates

As shown in Fig. 4 and Supplementary Fig. 1, the names and signatures of NLRC3 subfamily genes were chaotic in different species, especially in fish species. According to the relationships of NLRC3 genes in the phylogenetic analysis, the names of NLRC3 genes in black rockfish were normalized and standardized. Moreover, most NLRC3 genes of black rockfish clustered closely, which was similar for other fish species, especially honeycomb rockfish. Strikingly, not only were NLRC3 gene clusters for black rockfish gathered with several NLRC3 genes of other fish species, but also the clusters of NLRC3 genes in other fish species included certain members of NLRC3 expansions of black rockfish. In addition, several clusters of NLRC3 genes consisted of genes from all fish species. Interestingly, the NLRC3 cluster of high vertebrates had a close relationship with several NLRC3 genes of fish species.

Phylogenetic analysis of the NLRC3 genes in black rockfish and honeycomb rockfish, together with high vertebrates, showed that there were both species-specific NLRC3 expansions for each fish species, and correlated expansions of NLRC3 genes between fish species, as well as other high vertebrates (Supplementary Fig. 2). Furthermore, the NLRC3 genes of black rockfish were relatively separate and conservative compared to other species.

**Fig. 5:**
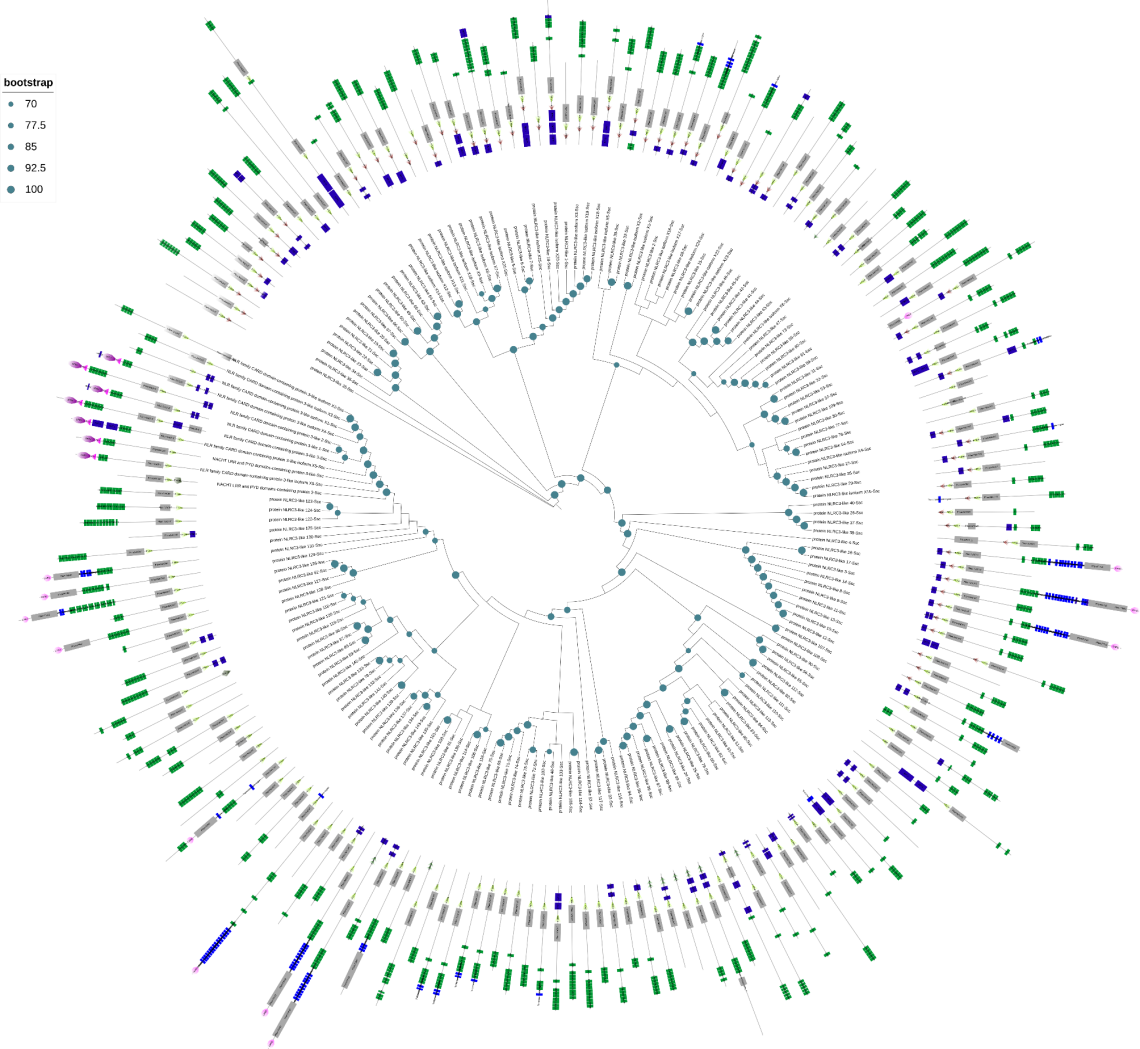
Phylogenetic tree of full NLRC3 contigs with associated protein domain architectures in black rockfish

Phylogenetic analyses of NLRC3 genes in black rockfish demonstrated strong phylogenetic support (Fig. 5). The FISNA domains existed in all NLRC3 gene architectures, together with protein domains of Pfam:NACHT, RING and Leucine-rich repeats (LRRs) (Fig. 5 and Supplementary Table 1). Nonetheless, the absence of LRRs were observed in several NLRC3 homologs and some homologs possessed other functional domains, such as PRY domains, SPRY domains and CARD domains. Moreover, an additional phylogenetic analysis using only FISNA domains from each NLRC3 gene of black rockfish to compare with full NLRC3 contigs (Supplementary Fig. 3). Similar aligning matrices of FISNA domain-only provided greater nodal support to the full NLRC3 contig tree in the phylogenetic relationship. Meanwhile, a bit of differently phylogenetic nodes revealed the specificities of FISNA domains in different NLRC3 genes.

### Functional enrichment analyses of differentially expressed NLRC3 genes

Differentially expressed NLRC3 genes in black rockfish spleen following challenge with *A. salmonicida*, in the intestine following *E. tarda* challenge and in the liver following challenge with *A. salmonicida*, at different time points (2 h, 12 and 24 h), are summarized in Supplementary Tables 2–4 and displayed using Volcano plots in Supplementary Fig. 4A-C. In addition, heatmap profiles displaying the expressed patterns of NLRC3 genes in these different organs following challenge infection are shown in Supplementary Fig. 5.

There were various kinds of functional GO terms with complicated regulatory network relationships in different organs infected by different bacteria (Supplementary Fig. 6). Even though the number of significantly enriched GO terms was totally different between each transcriptome (most in intestine followed by liver, and the least in spleen), several of the same GO terms with immune function, such as immune system process and inflammatory response, were significantly enriched in all three organs. Overall, although 179 duplicate genes were obtained in the NLRC3 subfamily of black rockfish, only 120 NLRC3 genes (including 46, 63 and 42 in spleen, liver and intestine, respectively) were annotated in different organs during bacterial infection. Among these, just 16 NLRC3 genes performed the same functions in all three organs, while 15 NLRC3 orthologues showed different functions in different organs. In addition, more than 59 NLRC3 genes were not annotated, which might indicate that these duplicated genes were non-functional (Supplementary Tables 2–4 and Supplementary Fig. 6). Furthermore, the results of the comparative analyses of black rockfish testis and ovary in different developing stages showed that 32 and 26 NLRC3 genes differentially expressed significantly in testis and ovary, respectively. These 59 non-functional NLRC3 genes were still not expressed in the sexual organs of black rockfish (Supplementary Fig. 7).

## Discussion

While it is known that the small number of NLR proteins in mammals are involved in immune defense and recognize PAMPs [45–47], there have been few studies of the larger number of NLR proteins found in taxa other than mammals [28, 48], and very little information is available on NLRC3 homologs in teleosts. In this study, black rockfish (Sebastidae: Perciformes) was used as an animal model to explore the anti-bacterial functions of NLRC3 genes in the immune system, based on phylogenetic and transcriptomic analyses.

Expansions and contractions of gene families in black rockfish were quite distinct from other fish species, even those in the same order and family. This may be a result of WGD events, as it appears that one less ancient duplication event occurred during the evolution of black rockfish than in its closely related congener, honeycomb rockfish. Previous studies have suggested that genome duplication has contributed to the diversity of the genome in vertebrates (including fishes) during evolution, by driving expansions and contractions of gene families [49, 50]. Following gene duplication, one of the genes may be lost (non-functionalization), both duplicate genes may be retained, acquiring changes to divide the functions of the ancestral gene among the duplicates (subfunctionalization), and finally one of them may acquire a new function (neofunctionalization) [51]. Based on the functional annotation of NLRC3 genes in different organs (Supplementary Fig. 6), the results of this study suggest that the large number of duplicate gene pairs of the NLRC3 subfamily in black rockfish may have undergone all these fates.

It has been reported that at least one WGD event occurred throughout the whole teleost lineage [52–54], and in this study, extreme expansions of NLRC3 homologs were found in teleost fishes compared to other vertebrates. Moreover, NLRC3 genes were identified as having key regulatory roles in several biological processes, which is consistent with the gene balance hypothesis (retention of regulatory genes following gene duplication) [49]. Furthermore, according to GO functional enrichment analyses, various functions were predicted for the NLRC3 genes in black rockfish, which might suggest differences between fishes and land vertebrates in gene retention for different functional categories of genes during evolution [49].

In particular, the reproductive process was enriched significantly among the GO terms of NLRC3 genes in black rockfish. Previous studies have documented that a subset of NLR genes are implicated in reproductive functions [55]. For the intestine, several developmental disorders (such as persistent trophoblastic disease) have been identified to be associated with mutations in NLR genes [56].For example, members of the reproduction-associated human NLR gene cluster are highly expressed in oocytes and ovaries [57] and several other members of the subset of NLR genes have also been found in all stages of follicle development [58, 59]. Findings in mammals have shown that reproduction-associated NLR genes do not perform any functions in immunity, and immune-related NLRs are not expressed at high levels in oocytes and ovaries [60]. By contrast, in black rockfish, our results showed that NLRC3 genes not only participated in the reproductive process with significantly differential expressions in the testis and ovary development, but also performed functions in several immune-related pathways, which might suggest the functional diversification of NLRC3 genes during teleost evolution. However, further studies are needed to verify this hypothesis.

Phylogenetic analyses displayed the conserved nature of NLRC3 homologues in black rockfish, which was similar to the situation in zebrafish [61]. The similar aligning matrices of FISNA domain-only to the full NLRC3 contig tree provided greater nodal support in the phylogenetic relationship. Meanwhile, a bit of differently phylogenetic nodes of FISNA domains also revealed the specificities of different NLRC3 genes in the same fish species. Furthermore, several NLRC3 genes in black rockfish, honeycomb rockfish and yellow perch, as well as zebrafish, showed close evolutionary relationships. Some NLRC3 genes of fish species were also clustered with higher vertebrate NLRC3 genes. This was also reported by Chang et al. who found that NLRC3 genes in zebrafish showed a close relationship with grass carp and human NLRC3 genes [39].

Architectural analyses of protein domains provided evidence for the origin and evolution of piscine specific NLRC3 genes. In black rockfish, almost all NLRC3 genes possessed a central NACHT domain that is highly similar to the NACHT domain of NLRA3 genes, which might suggest their evolution from a NLRA3-like molecule. Moreover, the PRY-SPRY domain existing in certain NLRC3 genes has also been found on several tripartite motif (TRIM) proteins and on the PYRIN molecule [62, 63]. TRIM5a has been reported to inhibit retroviral activity by binding to the capsid of the HIV retrovirus [64]. PYRIN may perform proinflammatory functions to protect against systemic infection by decreasing bacterial loads during infection [65]. Because of the similarity of the predicted N-and C-terminal structures of some NLRC3 genes to the domains of the PYRIN molecule, the potential functions of NLRC3 genes may mimic the functions of PYRIN. In addition, NLRC3 genes in black rockfish contained NACHT domains similar to NLRA3 genes that have inhibitory roles in T cells in mammals [66]. Therefore, NLRC3 genes may play important roles in the immune response of black rockfish during bacterial infection, although further studies are required to determine the detail of these roles. In addition, NLRC3 genes have been reported to suppress the inflammatory response by inhibiting NLRP3 inflammasome assembly [67, 68]. In teleosts, it has been reported that NLRC3 genes could be involved in the inflammatory response together with NLRP3 genes [69]. However, as shown in Fig. 3, the copy numbers of NLRC3 and NLRP3 genes were positively correlated with the loss of TLR4 genes. The loss of TLR4 genes in those fishes may relate to the expansion events of the NLRC3 genes in teleost fishes. Lost functions of TLR4 genes might be compensated for by expanded copies of NLRC3 genes.

Even though the antiviral roles of NLRC3 genes were reported in different fish species [44], such as *Lates calcarifer* [70], *M. miiuy* [26], etc., the antibacterial roles of piscine NLRC3 genes seemed to deserve a lot more attention and research in teleost, especially in black rockfish. Following infections with Gram-negative bacteria, different patterns of gene expression were found in different organs (spleen, intestine and liver) of black rockfish. In spleen following *A. salmonicida* infection, the number of up-regulated NLRC3 genes was similar to the number of down-regulated genes at each time point post-infection. The spleen has long been recognized as an important immune organ and plays a key role in the immune response against bacterial infection. A number of studies have also reported the functional importance NLRC3 genes in the spleen in the immune response. For example, NLRC3 genes contribute to thymic development but suppress the functions of CD4 + T cells in the spleen of mice during bacterial infection [71]. In teleosts, NLRC3 genes were highly expressed in sevenband grouper (*Epinephelus septemfasciatus*) spleen and attenuated the interferon response by impacting TRAF6/NF-κB activity and reducing IFN sensitivity, ISRE promoter activity, and IFN pathway gene expression after viral infection, but increased the inflammasome response and pro-inflammatory gene expression [72]. In our previous studies, NLRC3 genes in the spleen were also induced in response to bacterial infection of black rockfish and found to play a key role in a mRNA-miRNA-lncRNA regulatory network associated with the immune response [73].

In black rockfish intestine following *E. tarda* infection, up-regulated NLRC3 genes were found at all the time points post infection, with an increasing trend of up-regulation versus down-regulation occurring as the time post infection increased. These findings suggest that NLRC3 genes in the mucosal immune system play vital roles against bacterial challenge in teleosts. NLRC3 genes are activated by the stimulation of butyrate to protect the intestinal barrier in a GPR43-dependent manner in the colonic tissues of mice [74], and regulate cellular proliferation and apoptosis to suppress colorectal tumorigenesis [75, 76]. In teleosts, NLRC3 genes were also found to have high expression following different bacterial infections in the intestine of channel catfish [77], turbot [27, 78] and black rockfish [29].

In the liver, another well-recognized immune organ, a large proportion of NLRC3 genes were significantly up-regulated at all time points post infection in black rockfish during *A. salmonicida* challenge. NLRC3 genes were also up-regulated in the liver of rainbow trout following simulation with bacterial LPS for 12 h [40] and in the liver of channel catfish challenged by different bacteria at 12 h [77]. This up-regulation of NLRC3 genes occurred at early time points post bacterial infection, which might suggest that NLRC3 genes primarily played a negative regulatory role on the liver of black rockfish, suppressing the relevant hepatic immune system to reduce the inflammation response caused by bacterial challenge. A negative regulatory role of NLRC3 genes for the liver has previously been reported in hepatic diseases. For example, NLRC3 genes appear to reduce inflammation by inhibiting the activation of NF–κB signal pathway [79]. In addition, NLRC3 silencing may play an important role in cancer progression and prognosis of hepatocellular carcinoma via the activation of Janus kinase 2/signal transducers and the transcription 3 (JAK2/STAT3) pathway under interleukin-6 (IL-6) stimulation [80, 81].

## Conclusions

In the present study, phylogenetic analyses of 17 species have unraveled the evolutionary history of key immune genes and also revealed that the expansion events of NLRC3 genes during teleost evolution might have played a role in the functional diversification of this gene family. In addition, different patterns of expression of NLRC3 genes in black rockfish following different bacterial infections has suggested the immune function of NLRC3 genes in the traditional and mucosal immune system of the Sebastidae family. Overall, we hypothesize that different teleost lineages have evolved different patterns of functional diversities of NLRC3 genes following whole genome duplication events, but deeper functional studies are urgently needed to confirm this hypothesis. These findings provide a new starting point for further research into NLRC3 gene functions in the immune response and other processes in teleost fishes.

## Materials and methods

### Data set

Based on the distance of the evolutionary relationship to black rockfish in the taxonomy tree, 17 species were randomly chosen for the relevant analyses in this study. Subsequently, genome and protein sequence data for domestic chicken (*Gallus gallus*), Chinese softshell turtle (*Pelodiscus sinensis*), African clawed frog (*Xenopus laevis*) and 13 fish species, including zebrafish (*Danio rerio*), pike (*Esox lucius*), rainbow trout, mangrove killifish (*Kryptolebias marmoratus*), ballan wrasse (*Labrus bergylta*), large yellow croaker (*Larimichthys crocea*), giant grouper (*Epinephelus lanceolatus*), leopard coral grouper (*Plectropomus leopardus*), yellow perch (*Perca flavescens*), honeycomb rockfish, lumpfish (*Cyclopterus lumpus*), nine-spined stickleback (*Pungitius pungitius*), and three-spined stickleback (*Gasterosteus aculeatus*), were downloaded from the NCBI FIT site (https://ftp.ncbi.nlm.nih.gov/), while sequence data for black rockfish were provided by the Fish Immunology Laboratory of Qingdao Agricultural University, which also can be downloaded from the NCBI FIT site (see Supplementary Table 5 for a full list of submitter and RefSeq access numbers). The sequencing quality and assembly integrity of this genome data were identified and analyzed by the previous study [82], which indicated that this genome data can be used for the following analyses in this study. Taking the finished genome of each species as an input file, protein-coding gene sets were predicted by using the Prodigal progress package, using default parameters [83]. The predicted protein-coding gene sets were combined with the protein sequence data to determine the final protein sequence dataset in each species for subsequent analyses. In addition, the transcriptomic RNA-seq data of black rockfish were obtained from the following published studies: transcriptomic data of the spleen following *Aeromonas salmonicida* challenge [73], of the intestine following *Edwardsiella tarda* challenge [84], and of the liver following challenge by *A. salmonicida* [85].

Trinity progress packages were employed for de novo assembly of transcript sequences of black rockfish transcriptomic data for downstream analysis of gene expression [86]. Briefly, the RNA-seq data were assembled into unique sequences to generate full-length transcripts for a dominant isoform (contigs) by the Inchworm software module using the k-mer algorithm. Next, in the Chrysalis software module, the contigs generated in the Inchworm module were clustered and complete de Bruijn graphs for each cluster were constructed. Finally, the individual graphs in parallel were processed in the Butterfly module to trace the paths of reads and pairs of reads in the graphs, report full-length transcript sequences for alternatively spliced isoforms, and tease apart transcripts corresponding to paralogous genes.

### Multiple sequence alignment and phylogeny reconstruction

Orthologues were inferred by taking the proteome data of the 17 species (Supplementary Table 5) through OrthoFinder v.2.5.4, a software program for the inference of phylogenetic orthology [87, 88]. The single-copy orthologue protein sequences were obtained and used to create a large contiguous sequence for each species by Python package and scripts. Then, multiple sequence alignments of these large contiguous sequences were conducted using the MAFFT v.7.505 program [89]. IQ-TREE v.2.2.0.3 was employed to calculate the best-fit substitution model by computing the log-likelihoods of an initial parsimony tree for many different models based on the Akaike information criterion (AIC), corrected Akaike information criterion (AICc), and the Bayesian information criterion (BIC) [90], and an outgroup taxon was predicted for drawing a maximum-likelihood ultrametric species tree [91]. Maximum-likelihood phylogenetic construction was performed with RAxML-NG v.1.1.0) using the best-fit substitution model (JTT + FC + R4), with *Gallus gallus* used as the outgroup taxon [92]. The standard non-parametric bootstrap criterion was set to determine the optimal number of bootstrap replicates [93, 94]. The catalogue information of fish species were obtained from FishBase [95].

### Expansions and contractions of gene families

Nucleotide sequences were obtained from NCBI databases using Python scripts, based on the protein access numbers of single copy orthologue sequences. The large contiguous nucleotide sequences were also created by the above method. Multiple alignments of nucleotide sequences were performed with the MAFFT program, and then converted into PAML format by the EasyCodeML program [96]. The phylogenetic tree in Newick format of 17 species with fossil record calibrations was obtained to determine approximate likelihood calculations, based on data from the TimeTree online website [97]. The ultrametric species tree with divergence time inferences was estimated using the MCMCTree v.4.9 program by inputting multiple nucleotide sequence alignments, the phylogenetic tree of 17 species with fossil calibrations, and a control file containing the instructions for this program [98]. The parameters of the control file were set as follows: ‘clock = 2’, ‘model = 4’, ‘alpha = 0’, ‘ncatG = 5’, ‘cleandata = 0’, ‘BDparas = 1 1 0’, ‘kappa_gamma = 6 2’, ‘alpha_gamma = 1 1’, ‘rgene_gamma = 2 2’, ‘sigma2_gamma = 1 10’, ‘finetune = 1: 0.1 0.1 0.1 0.01 .5’, and ‘print = 1’. All other parameters were set to default.

The data of orthologues and orthogroups in the proteomes were used from the output results of OrthoFinder v.2.5.4 above. The longest protein sequence of each orthologue was used to identify gene duplication events following the instruction of OrthoFinder. Duplication ratios per node/tip were calculated by dividing the number of duplications observed in each node/tip by the total number of gene trees containing that node/tip. The gene family counts identified by OrthoFinder and a rooted, binary, and ultrametric species tree in Newick format from the output of the MCMCTree analysis were then taken as input files to perform iterative runs of the likelihood-based method in CAFE5 software to infer rates of gene expansions and contractions [99]. Gene expansions and contractions were then assessed by regressing gene counts at nodes Vstips of the tree, and the expansion/contraction within each gene family with *P*-value < 0.05 were assessed as statistically significant.

### Whole genome duplication analyses

Whole genome duplication analyses were performed by WGDI v.0.5.6, a Python-based command-line tool [100]. Genome, CDS, protein and GFF3 data from black rockfish and honeycomb rockfish were processed into input files for subsequent WGDI analyses. Then, to assess genome conservation and duplication events, intraspecific synteny, collinearity and synonymous substitutions per site (Ks) were analyzed and estimated. Finally, dot plots and frequency distributions of the black rockfish and honeycomb rockfish were drawn using python packages in WGDI.

### Copy number estimation of NLRC3 genes

To estimate the number of copies of NLRC3 genes in each of the selected species, conserved FISNA domains were used as the query sequences. The FISNA sequences of part members of NLRC3 genes in seven fish species, including black rockfish together with lumpfish, yellow perch, nine-spined stickleback, pike, zebrafish and honeycomb rockfish, were extracted and collected (Supplementary Table 6). NLRC3 target sequences were prepared and obtained through all hits detected in the individual proteomic database of each of 17 species with the BLASTP program (the cutoff setting of e-value is 1 × e^− 5^) using FISNA domain sequences from the seven teleost reference proteomes as queries. The number of target gene copies of each species was determined on the basis of the number of unique reads matching these FISNA domains against each of the reference gene regions in proteomes. Finally, the copy numbers of each target gene were estimated through mapping these unique reads to the nr database (http://://ftp.ncbi.nlm.nih.gov/blast/db/FASTA/) and conserved domain database (CDD) in the NCBI website [101]. The nucleotide and protein sequences of black rockfish NLRC3 subfamily homologues were extracted from the CDS and protein databases of black rockfish and confirmed by referencing to its genomic database. Python scripts and codes were edited to remove the reduplicated sequences.

To assess the functions of NLRC3 genes, the immune gene repertoires of the teleost genomes, including several conserved immune genes and several genes interacting with NLRC3 genes, were investigated through a comparative gene mining pipeline comprising BLAST searches, prediction of ORFs and annotation. AP1M2 (adaptor related protein complex 1 subunit mu 2), AP2M1 (adaptor related protein complex 2 subunit mu 1), AP3M2 (adaptor related protein complex 3 subunit mu 2), RIPK2 (receptor interacting serine threonine kinase 2), TLR4 (toll-like receptor 4), TLR9 (toll-like receptor 9), TRAF6 (TNF receptor associated factor 6) and NLRP3 (NACHT, LRR and PYD domains-containing protein 3) were confirmed. Moreover, two highly conserved genes (RAG1: recombination activating gene 1, and RAG2: recombination activating gene 2) were included as controls. Then, the orthologue sequences of these selected genes for nine species, including chicken, Chinese softshell turtle, African clawed frog, lumpfish, yellow perch, nine-spined stickleback, pike, zebrafish and honeycomb rockfish, were collected as queries to determine the copy numbers of these genes in all 17 species using the above methodology.

### Phylogenetic analyses of NLRC3 subfamily

To evaluate the phylogenetic orthology of the NLRC3 subfamily, NLRC3 protein sequences of the 14 selected fish species and other high vertebrates (chicken, turtle and frog) were aligned using the MAFFT v.7.505 program. The best-fit substitution model (JTT + F + I + G4) was calculated by IQ-TREE v.2.2.0.3 and a maximum-likelihood phylogenetic tree was constructed on RAxML-NG v.1.1.0. The NLRC3 subfamily sequences of black rockfish were defined and annotated by the phylogenetic relationships between black rockfish and other species and the protein domain architecture on CDD and SMART databases [102]. Two additional phylogenetic analyses were run, using the sequences of NLRC3 genes of black rockfish, honeycomb rockfish and high vertebrates, and only the FISNA domains of the black rockfish to compare the NLRC3 orthology inference. The iTOL online tool was employed to modify the final phylogenetic trees [103].

### Functional enrichment of differentially expressed NLRC3 genes

The data for differentially expressed genes of the black rockfish spleen challenged by *A. salmonicida*, intestine infected by *E. tarda* and liver with *A. salmonicida*, at different time points post infection (2 h, 12 and 24 h) were obtained from previous studies [73, 84, 85]. Differential expression analysis of the transcripts was performed using StringTie software and the DESeq R package (3.0.3) [104, 105]. Reads per kilobase of transcript per million mapped reads (RPKM) were obtained. The Benjamini-Hochberg correction procedure was used to adjust the resulting *P*-value for false discovery rate [106]. The expressed level of transcripts with | log2(Fold Change) | > 0 and *P*-value < 0.05 were assigned as differentially expressed. Subsequently, based on NLRC3 target sequences of the black rockfish determined above, differential expression patterns for NLRC3 genes were obtained and displayed using Heatmap and volcano plots on the web server of ImageGP [107]. In addition, the transcriptomic data sets of different organs of black rockfish, such as testis and ovary sexual organs in different developing stages (PRJNA573572), were downloaded and analyzed in the same methods descripted above. The Heatmap plots of NLRC3 genes in these organs were drawn to support the relevant findings in this study.

In order to verify the functional processes in which differentially expressed NLRC3 genes in the black rockfish participate, Gene Ontology (GO) analyses were performed using Blast2GO v.6.0.3 software via a series of analysis schemes [108]. In detail, the query sequences of differentially expressed NLRC3 genes in the three black rockfish transcriptomic databases were matched against the GO annotation database with GO mapping after running BLAST at the NCBI. Next, functional annotation was conducted to select GO terms from the GO pool obtained by the mapping step and assign them to the query sequences. Then, GO-slim was carried out to functionally summarize a sequence dataset in a uniform and species-specific way. Finally, GO graph visualization was used to generate combined gene ontology annotation graphs in three GO functional categories (Biological Process, Molecular Function, Cellular Component).

### Electronic supplementary material

Below is the link to the electronic supplementary material.

Supplementary Material 1

Supplementary Material 2

Supplementary Material 3

Supplementary Material 4

Supplementary Material 5

Supplementary Material 6

Supplementary Material 7

Supplementary Material 8

Supplementary Material 9

Supplementary Material 10

Supplementary Material 11

Supplementary Material 12

Supplementary Material 13

Supplementary Material 14

## Data Availability

The raw data of transcriptome and genome of black rockfish supporting the conclusions of this article were made available by the authors, without undue reservation. The datasets of black rockfish and other species presented in this study can be also found in online repositories (https://www.ncbi.nlm.nih.gov/genome/gdv/). The names of the repository/repositories and accession number(s) can be found in the article/Supplementary Material. The sequences of all NLRC3 genes mentioned in this article were submitted to the GeneBank (https://www.ncbi.nlm.nih.gov/genbank/), and the accession numbers of these NLRC3 sequences are OQ857571 - OQ857749.
