# Supplementary figures and images for: The evolution of NLRC3 subfamily genes in Sebastidae teleost fishes
Source: BMC Genomics. 2023 Nov 14;24:683. doi: 10.1186/s12864-023-09785-5 (PMC10648357; doi:10.1186/s12864-023-09785-5)

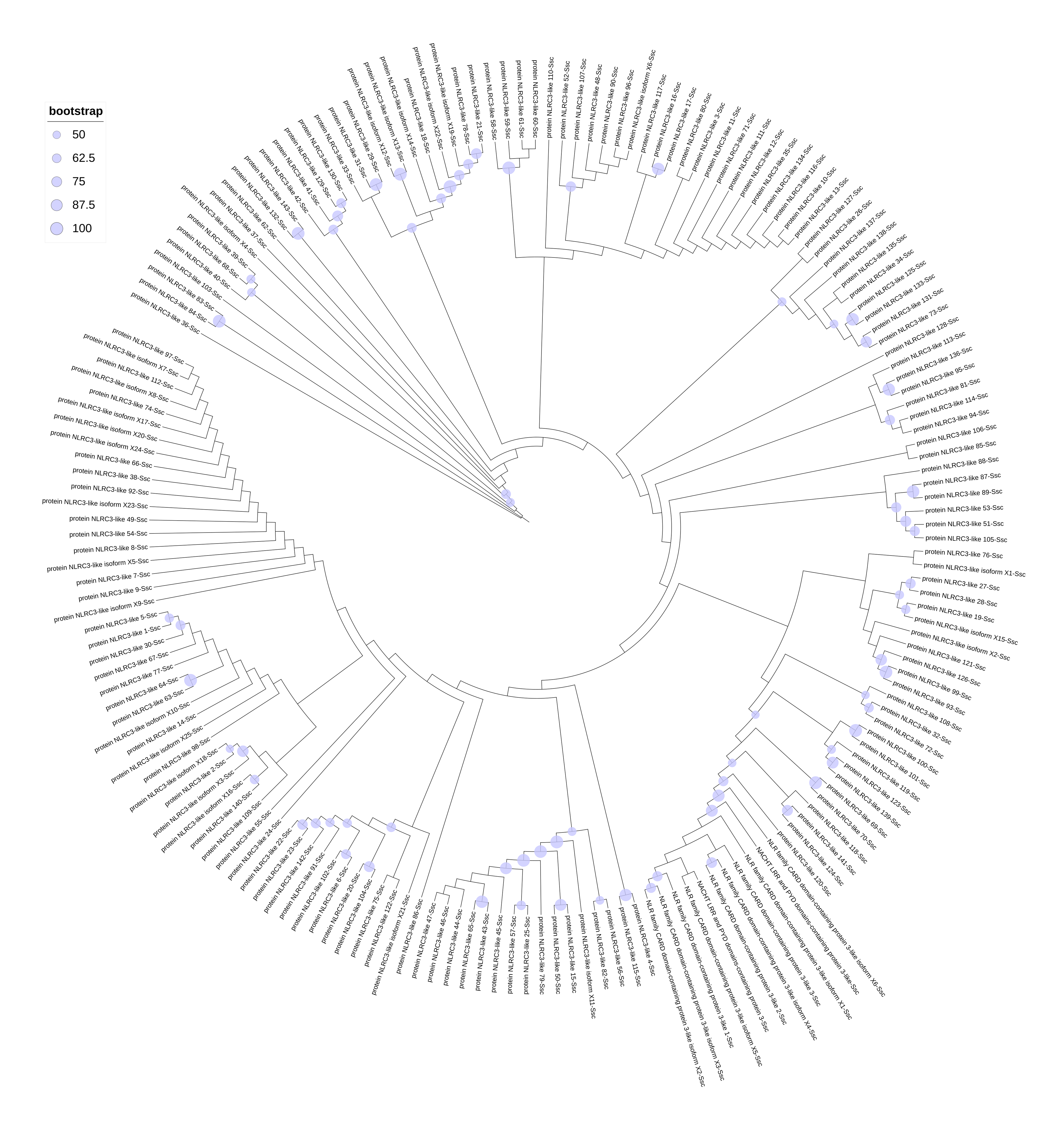

Supplement: Supplementary file 2 — Supplementary Material 2 [file 12864_2023_9785_MOESM2_ESM.tif]

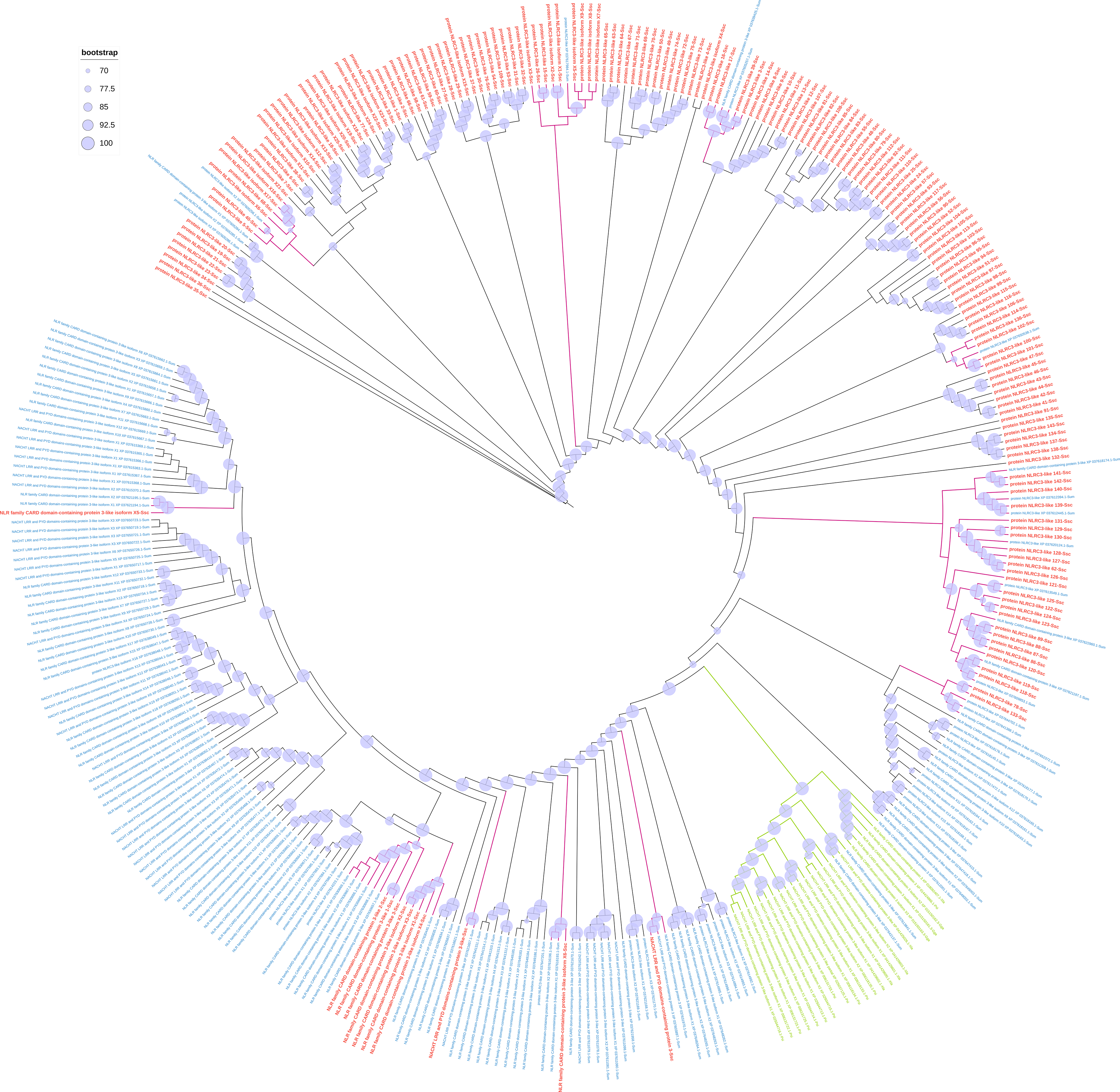

Supplement: Supplementary file 4 — Supplementary Material 4 [file 12864_2023_9785_MOESM4_ESM.tif]

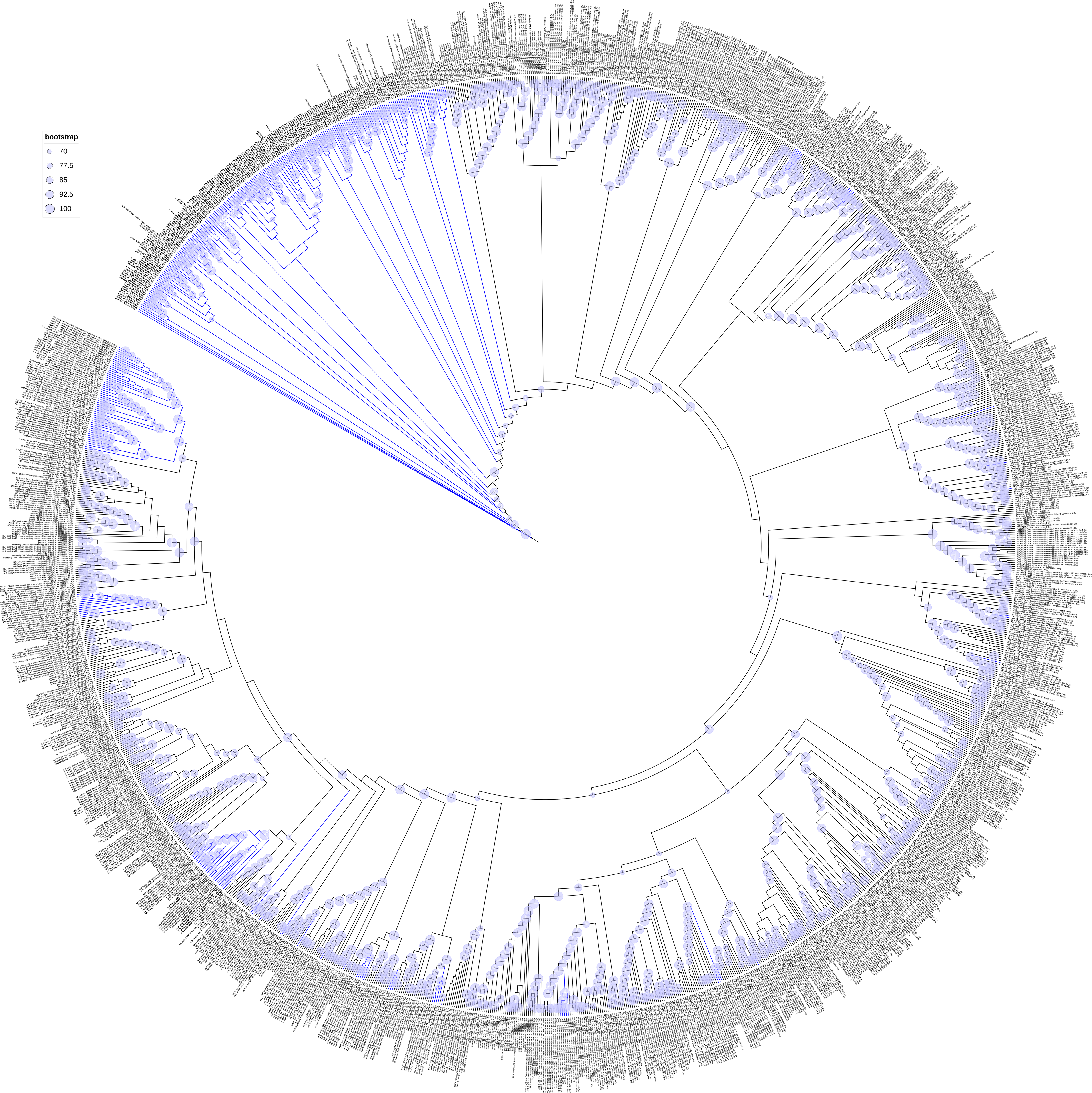

Supplement: Supplementary file 6 — Supplementary Material 6 [file 12864_2023_9785_MOESM6_ESM.tif]

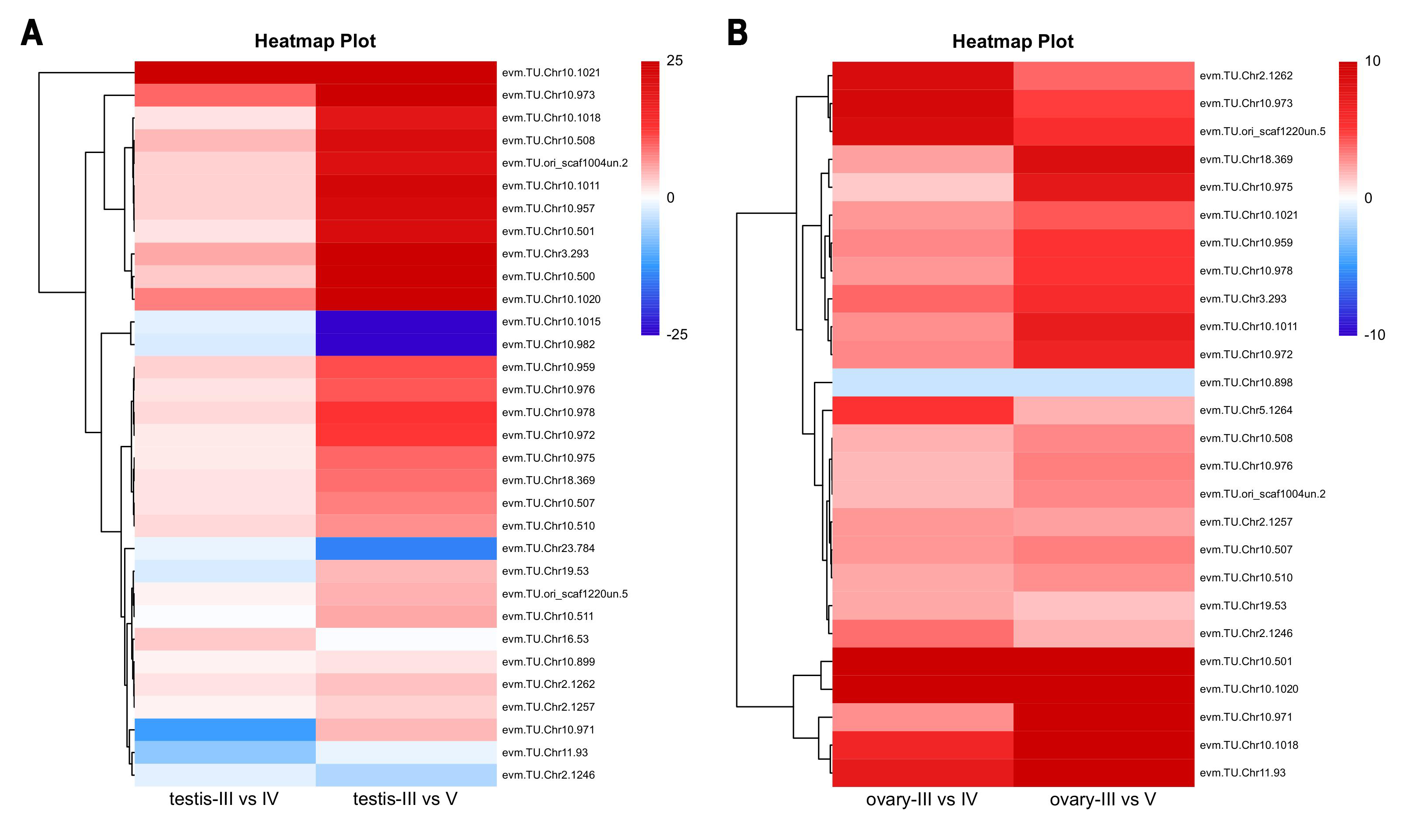

Supplement: Supplementary file 7 — Supplementary Material 7 [file 12864_2023_9785_MOESM7_ESM.tif]
