# Supplementary material for: The evolution of NLRC3 subfamily genes in Sebastidae teleost fishes
Source: BMC Genomics. 2023 Nov 14;24:683. doi: 10.1186/s12864-023-09785-5 (PMC10648357; doi:10.1186/s12864-023-09785-5)
Supplement: Supplementary file 8 — Supplementary Material 8 [file 12864_2023_9785_MOESM8_ESM.docx]

**Supplementary Fig 1**. Phylogenetic tree of NLRC3 homologs in the 14 selected fish species and high vertebrates. NLRC3 genes of black rockfish in bold font. Blue branches indicate NLRC3 expansion in the Sebastidae.

**Supplementary Fig 2.** Phylogenetic tree of NLRC3 homologs of Sebastidae species and high vertebrates. NLRC3 genes of the black rockfish were in bold font. Red and blue labels indicate lineage-specific NLRC3 clusters in black rockfish and honeycomb rockfish, respectively. Geranium pink branches indicate clusters of NLRC3 genes in both black rockfish and honeycomb rockfish, and the green cluster for high vertebrates.

**Supplementary Fig 3**. Phylogenetic tree of FISNA domain-only in black rockfish.

**Supplementary Fig 4**. Volcano plots of differentially expressed NLRC3 genes in black rockfish following different bacterial infections. (A) Differentially expressed NLRC3 genes in black rockfish spleen following *A. salmonicida* infection. (B) Differentially expressed NLRC3 genes in black rockfish intestine following *E. tarda* infection. (C) Differentially expressed NLRC3 genes in black rockfish liver following *A. salmonicida* infection. Dots in red and green represent significantly up-regulated and down-regulated genes, respectively. Gray dots represent genes with non-significant differential expression.

**Supplementary Fig 5**. Heatmaps of differentially expressed NLRC3 genes in black rockfish following different bacterial infection. (A) Differentially expressed NLRC3 genes in black rockfish spleen following *A. salmonicida* infection. (B) Differentially expressed NLRC3 genes in the black rockfish intestine following *E. tarda* infection. (C) Differentially expressed NLRC3 genes in the black rockfish liver following *A. salmonicida* infection. (a) All NLRC3 genes with significantly differential expression at least one time point post infection. (b) NLRC3 genes with significantly differential expression at all three time points post infection. Intensity of expression increases from blue to red. Each column represents one time point, and each row represents one gene.

**Supplementary Fig 6**. GO analyses of all differentially expressed NLRC3 genes in black rockfish spleen (A), intestine (B) and liver (C).

**Supplementary Fig 7.** Heatmaps of differentially expressed NLRC3 genes in black rockfish sexual organs in different developing stages. (A) Differentially expressed NLRC3 genes in black rockfish testis. (B) Differentially expressed NLRC3 genes in the black rockfish ovary. Intensity of expression increases from blue to red. Each column represents one time point, and each row represents one gene.
